# Supplementary material for: Tau, amyloid-β and α-synuclein co-pathologies synergistically enhance neuroinflammation and neuropathology
Source: bioRxiv. 2025 Nov 24:2024.10.13.618101. Preprint. [Version 3] doi: 10.1101/2024.10.13.618101 (PMC12424655; doi:10.1101/2024.10.13.618101)
Supplement: 1 [file NIHPP2024.10.13.618101V3-supplement-1.pdf]

bioRxiv preprint doi: https://doi.org/10.1101/2024.10.13.618101; this version posted November 24, 2025. The copyright holder for this preprint (which was not certified by peer review) is the author/funder, who has granted bioRxiv a license to display the preprint in perpetuity. It is made available under aCC-BY 4.0 International license.

Supplemental Figures

bioRxiv preprint doi: https://doi.org/10.1101/2024.10.13.618101; this version posted November 24, 2025. The copyright holder for this preprint (which was not certified by peer review) is the author/funder, who has granted bioRxiv a license to display the preprint in perpetuity. It is made available under aCC-BY 4.0 International license.

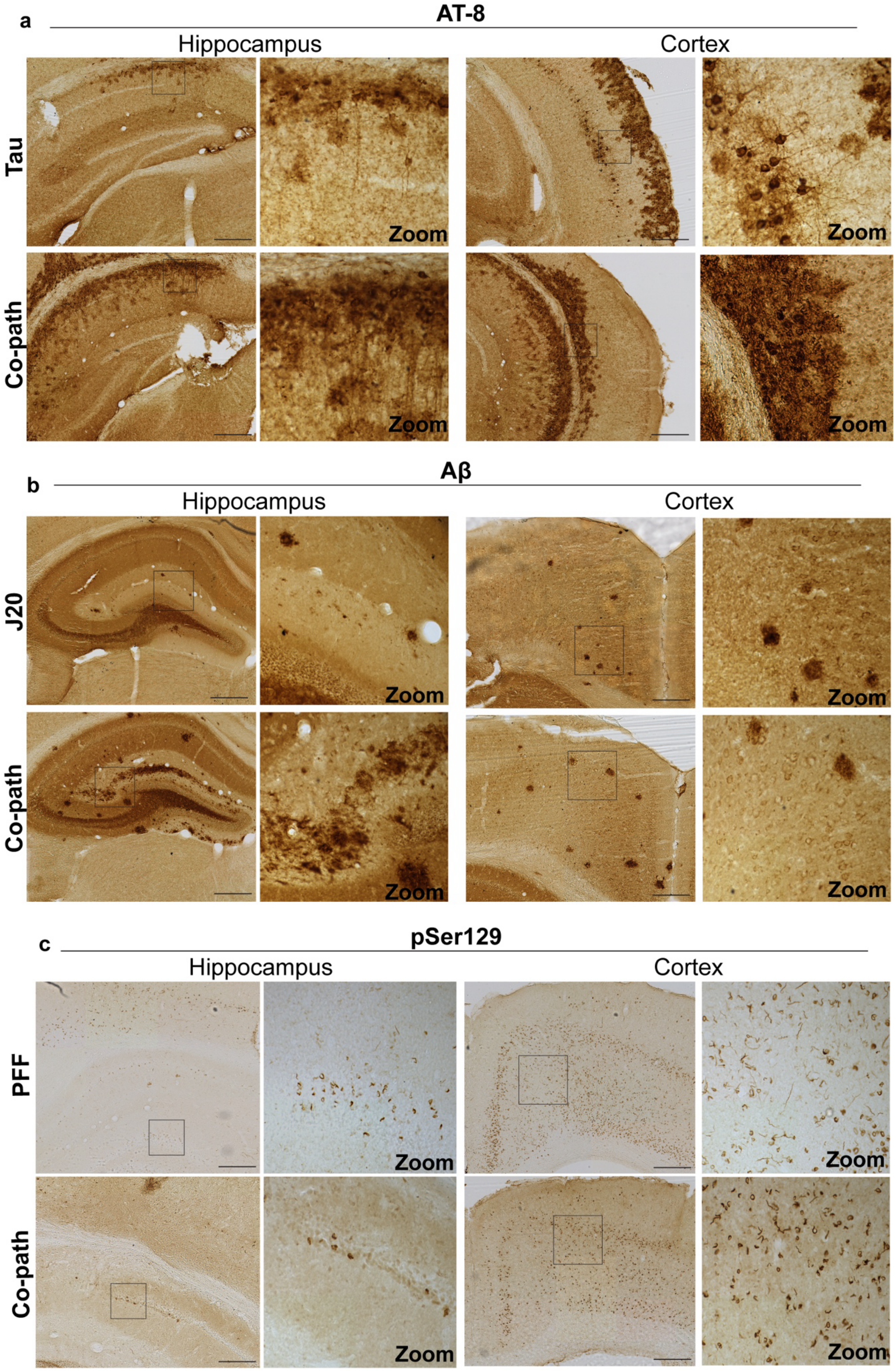

1339

bioRxiv preprint doi: https://doi.org/10.1101/2024.10.13.618101; this version posted November 24, 2025. The copyright holder for this preprint (which was not certified by peer review) is the author/funder, who has granted bioRxiv a license to display the preprint in perpetuity. It is made available under aCC-BY 4.0 International license.

Supplemental Figure 1. AT-8, Aβ or pSer129 protein pathology at 6MPI in the hippocampus and cortex of co-pathology and single pathology brains. (a) AT-8/pTau-positive neurons (DAB+, brown) in the hippocampus and cortex of tau single pathology and co-pathology brains. (b) Aβ-positive plaques (DAB+, brown) expressed in the hippocampus and cortex of J20 single pathology and co-pathology brains. (c) pSer120-positive inclusions in the hippocampus and cortex of PFF only (α-syn) and co-pathology brains. All images taken at 10X or 20X for zooms, scale bars = 100µm.

bioRxiv preprint doi: https://doi.org/10.1101/2024.10.13.618101; this version posted November 24, 2025. The copyright holder for this preprint (which was not certified by peer review) is the author/funder, who has granted bioRxiv a license to display the preprint in perpetuity. It is made available under aCC-BY 4.0 International license.

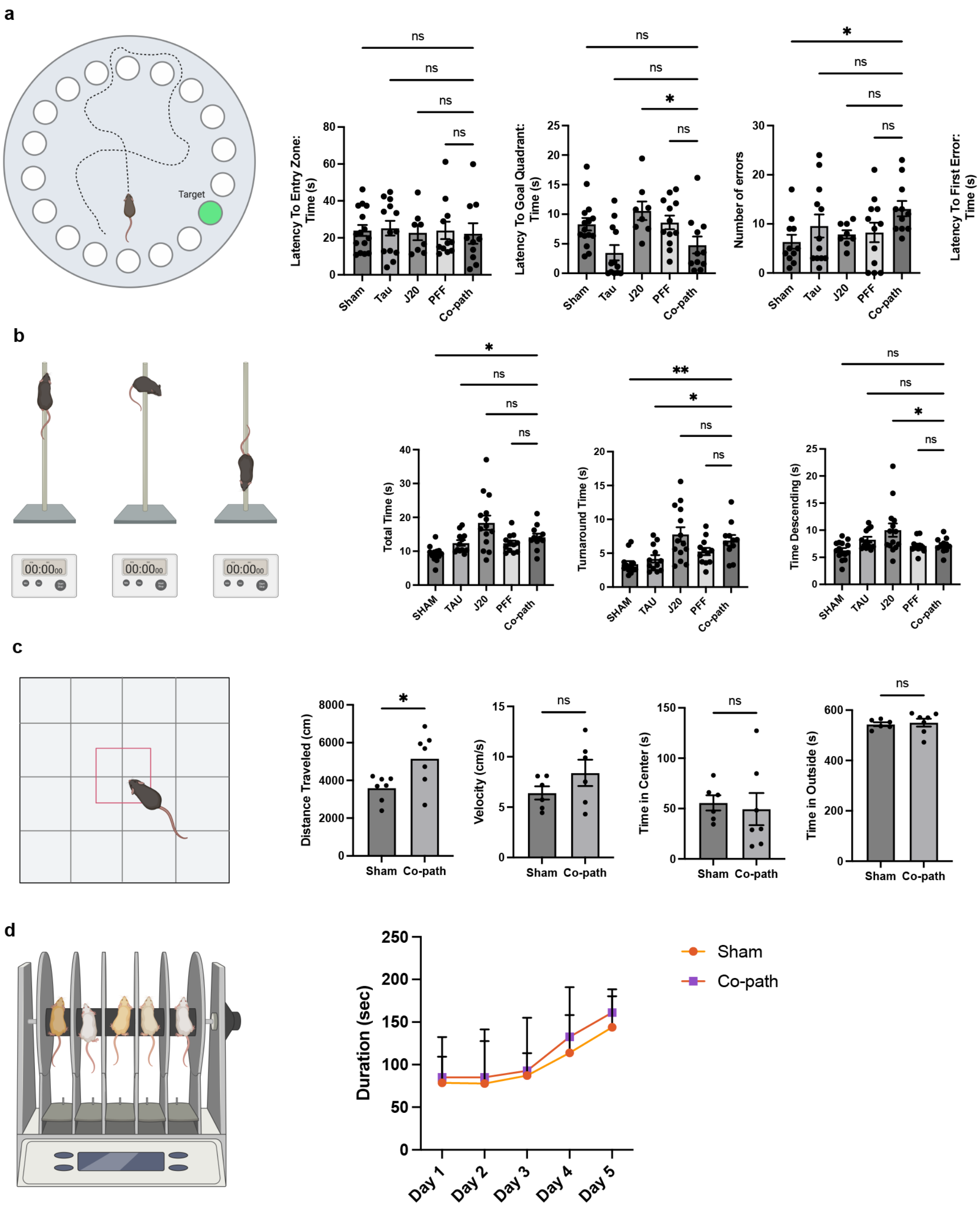

Supplemental Figure 2. Motor and cognitive behavioral tests to assess for impairments in co-pathology vs single pathology animals. (a) Quantification of latency to entry zone, to goal quadrant, to first error and the total number of errors analyzed from Barnes maze test. (b) Measures of total time, turnaround time and time descending on the pole test. (c) Distance travelled, velocity, time in center and time in outside are depicted from open field test. (d) Total duration of animals on rotarod platform across 5 days of testing. Student's t-test or One-way ANOVA with post hoc

bioRxiv preprint doi: https://doi.org/10.1101/2024.10.13.618101; this version posted November 24, 2025. The copyright holder for this preprint (which was not certified by peer review) is the author/funder, who has granted bioRxiv a license to display the preprint in perpetuity. It is made available under aCC-BY 4.0 International license.

for significance. Mean values +/- SEM are plotted. ns = no significance, *p<0.05, **p<0.01, ***p<0.005. n=8-10 animals per group, both males and females used.

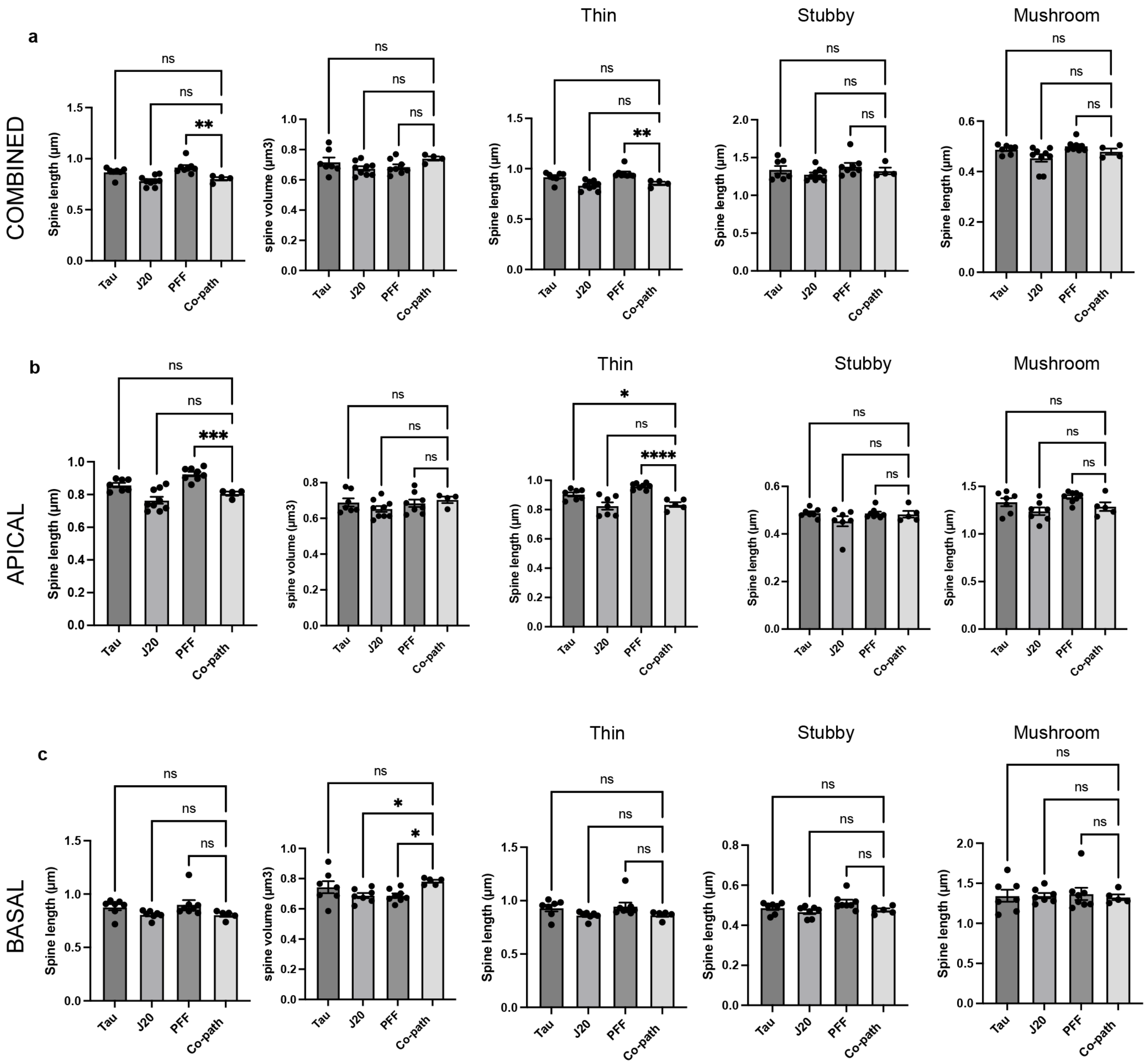

Supplemental Figure 3. Assessment of synapse integrity in the hippocampus of co-pathology and single pathology mice at 6MPI. Dendritic spine morphology was assessed in the CA1 region of hippocampus on pyramidal neurons and measures of total spine length and spine volume for all spines and for thin, stubby and mushroom subgroups are plotted from (a) combined (both apical

bioRxiv preprint doi: https://doi.org/10.1101/2024.10.13.618101; this version posted November 24, 2025. The copyright holder for this preprint (which was not certified by peer review) is the author/funder, who has granted bioRxiv a license to display the preprint in perpetuity. It is made available under aCC-BY 4.0 International license.

and basal), (b) apical only or (c) basal only dendrites. One-way ANOVA with post hoc for significance. Mean values +/- SEM are plotted. ns = no significance, *p<0.05, **p<0.01, ***p<0.005. n=8-10 animals per group, both males and females used.

bioRxiv preprint doi: https://doi.org/10.1101/2024.10.13.618101; this version posted November 24, 2025. The copyright holder for this preprint (which was not certified by peer review) is the author/funder, who has granted bioRxiv a license to display the preprint in perpetuity. It is made available under aCC-BY 4.0 International license.

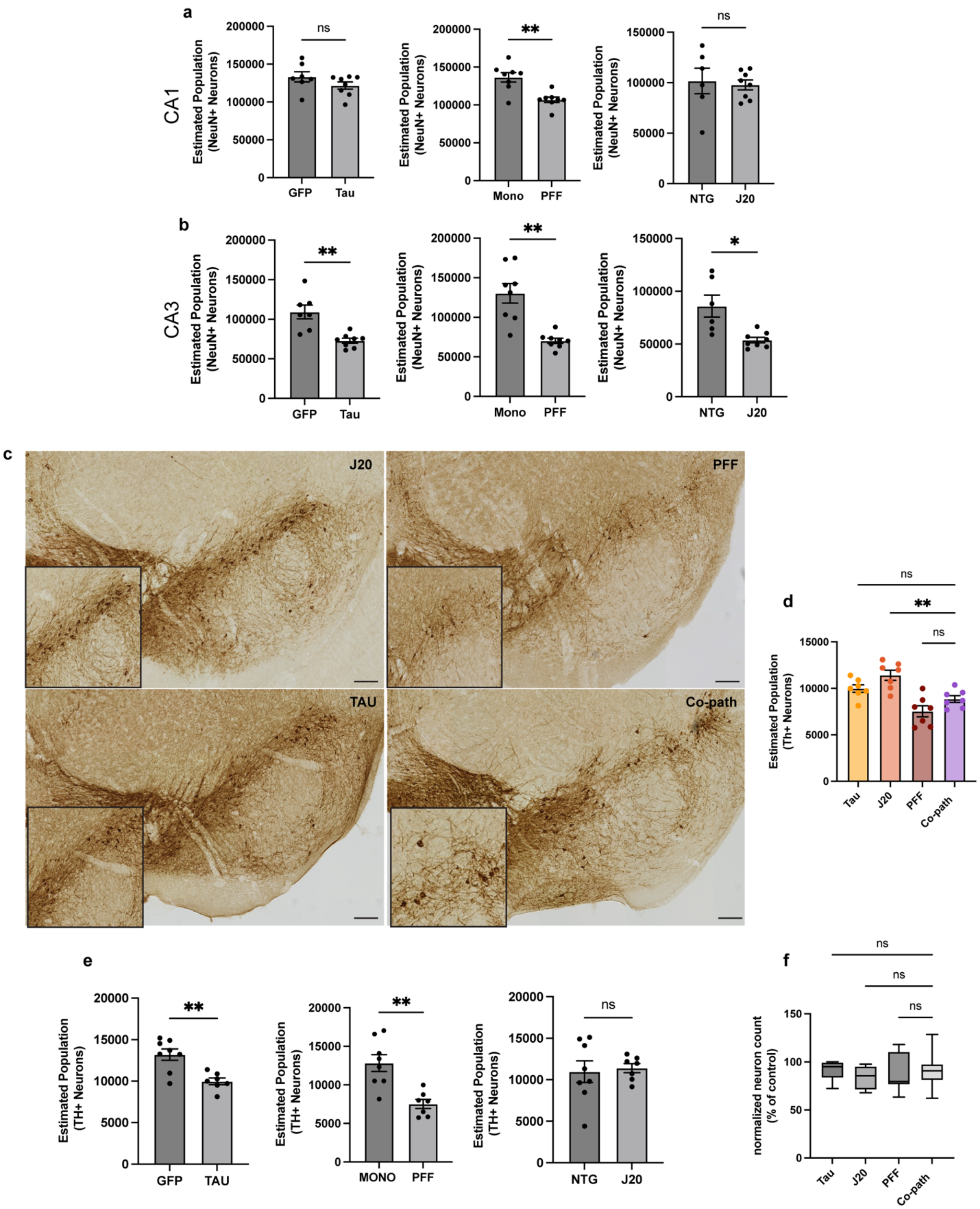

1370 **Supplemental Figure 4. Unbiased stereology for quantification of NeuN+ neurons in the**

1371 **hippocampus (single pathology and controls) and Tyrosine Hydroxylase positive neurons in**

bioRxiv preprint doi: https://doi.org/10.1101/2024.10.13.618101; this version posted November 24, 2025. The copyright holder for this preprint (which was not certified by peer review) is the author/funder, who has granted bioRxiv a license to display the preprint in perpetuity. It is made available under aCC-BY 4.0 International license.

the substantia nigra pars compacta (SNpc) at 6MPI. (a,b) Estimated population of NeuN+ neurons of co-pathology and single pathology CA1 or CA3 compared to their individual controls. (c) TH+ neurons (brown) in the SNpc of single pathology and co-pathology brains. (d) Unbiased stereology was used to quantify the number of TH+ neurons in the SNpc in the co-pathology mouse model compared to Tau, J20 and PFF single pathology brains. All images taken at 20X. Scale bars = 100µm. Student's t-test or One-way ANOVA with post hoc for significance. Mean values +/- SEM are plotted. Ns = no significance, **p<0.01, ***p<0.005, ****p<0.0001. n=7-8 animals per group, both males and females used.

bioRxiv preprint doi: https://doi.org/10.1101/2024.10.13.618101; this version posted November 24, 2025. The copyright holder for this preprint (which was not certified by peer review) is the author/funder, who has granted bioRxiv a license to display the preprint in perpetuity. It is made available under aCC-BY 4.0 International license.

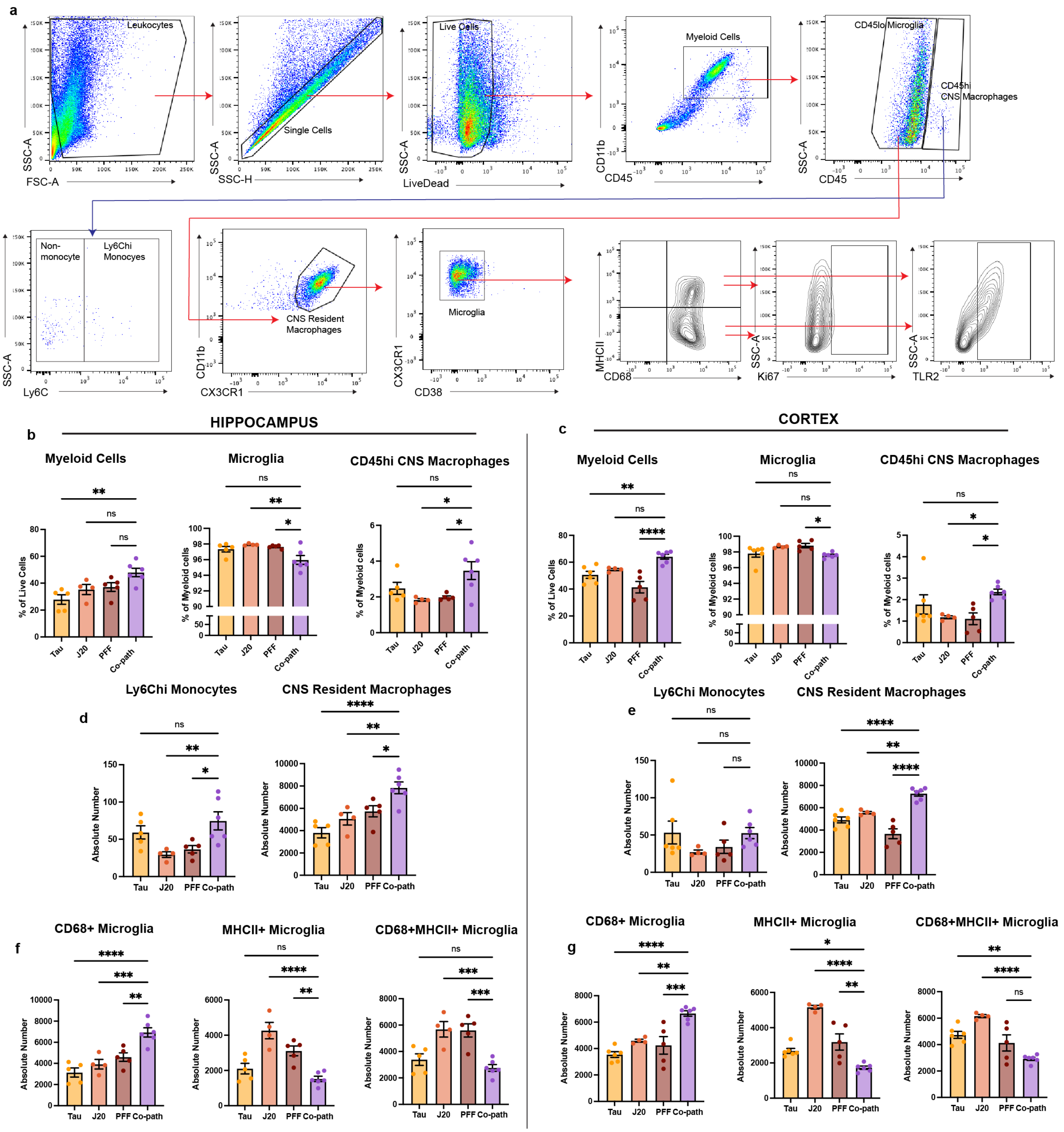

Supplemental Figure 5. Myeloid cell flow cytometry gating, validation and quantification in co-pathology and single pathology animals. (a) Gating strategy for flow cytometry analysis of microglia. (b) Quantification of proportion of myeloid cells, microglia and infiltrating

bioRxiv preprint doi: https://doi.org/10.1101/2024.10.13.618101; this version posted November 24, 2025. The copyright holder for this preprint (which was not certified by peer review) is the author/funder, who has granted bioRxiv a license to display the preprint in perpetuity. It is made available under aCC-BY 4.0 International license.

monocytes/CNS resident macrophages in the co-pathology model hippocampus and (c) cortex compared to single pathology brains. (d,e) absolute number of CD68+ microglia, MHCII+ microglia and MHCII+CD68+ microglia in the hippocampus and cortex of single pathology and co-pathology brains. Analyzed using One-way ANOVA. Mean values +/- SEM are plotted. *p<0.05, **p<0.01, ***p<0.005. n=5-6/group, with two brain regions pooled per sampled, both males and females included in analysis.

bioRxiv preprint doi: https://doi.org/10.1101/2024.10.13.618101; this version posted November 24, 2025. The copyright holder for this preprint (which was not certified by peer review) is the author/funder, who has granted bioRxiv a license to display the preprint in perpetuity. It is made available under aCC-BY 4.0 International license.

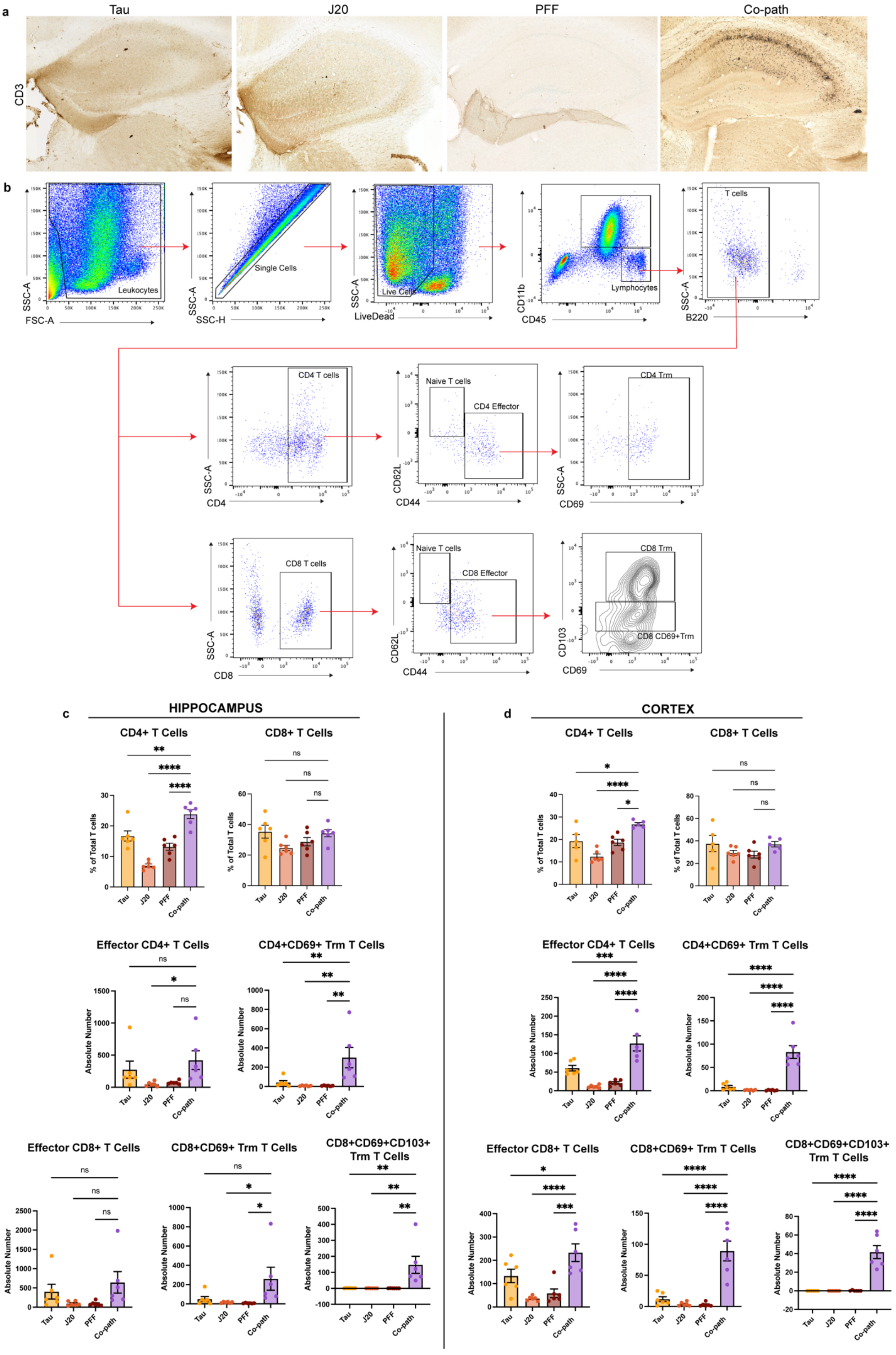

bioRxiv preprint doi: https://doi.org/10.1101/2024.10.13.618101; this version posted November 24, 2025. The copyright holder for this preprint (which was not certified by peer review) is the author/funder, who has granted bioRxiv a license to display the preprint in perpetuity. It is made available under aCC-BY 4.0 International license.

Supplemental Figure 6. T cell flow cytometry gating, validation and quantification in co-pathology and single pathology animals. (a) Representative images showing CD3+ staining (nickel) from Tau, J20, PFF and co-pathology brains. (b) Gating strategy for flow cytometry analysis of T cells. (c) Quantification from flow cytometry of proportion of CD4 and CD8 T cells, and absolute number of effector CD4 and CD8 T cells, and Trm T cells in the hippocampus and (d) cortex. Analyzed using One-way ANOVA. Mean values +/- SEM are plotted. *p<0.05, **p<0.01, ***p<0.005. n=5-6/group, with two brain regions pooled per sampled, both males and females included in analysis.
